# Composting Restructures Chicken Manure Viral Communities and Attenuates Virus-Associated Antibiotic Resistance Signals: Paired Metagenome and Virome Analyses

**DOI:** 10.3390/v18070789

**Published:** 2026-07-19

**Authors:** Weipeng Liu, Yongfeng Wang, Jian Ma, Xiaolong Liang, Liqiong Yang

**Affiliations:** 1 Institute of Applied Ecology, Chinese Academy of Sciences, Shenyang 110016, China; liuwp@iae.ac.cn (W.L.); mroger@163.com (J.M.); 2 CAS Key Laboratory of Forest Ecology and Silviculture, Institute of Applied Ecology, Chinese Academy of Sciences, Shenyang 110016, China; yfwang@iae.ac.cn; 3 University of Chinese Academy of Sciences, Beijing 100049, China

**Keywords:** chicken manure, composting, DNA virus, antibiotic resistance genes, viral particle enrichment, virus–host linkages

## Abstract

Composting is widely used to reduce biological risks during manure recycling, but changes in viral communities and virus-associated antibiotic resistance genes (ARGs) remain poorly resolved. This study aimed to assess changes in DNA viral communities, eukaryotic viral protein signals, virus-associated ARGs, and predicted virus–host linkages during chicken manure composting using paired analyses of total-community metagenomes and viral-particle-enriched viromes. Both approaches recovered viral assemblages dominated by Uroviricota and lytic viruses but produced distinct profiles. Viromes yielded more taxonomically assigned viral operational taxonomic units and a higher proportion of relatively complete viral genomes, whereas metagenomes produced a larger predicted virus–host network. Composting restructured viral communities, reducing manure-associated genera and enriching stage-specific groups. Eukaryotic viral protein signals declined during composting. Virus-associated ARGs accounted for 24.83–38.76% of ARG abundance in metagenomes and 5.38–45.09% in viromes, with lower abundance and richness in viromes. Selected virus-associated ARGs showed transient early enrichment, particularly in the virome. By maturity, both overall virus-associated ARG signals and higher-risk ARG signals had declined. Predicted virus–host associations included bacterial groups containing potential opportunistic pathogens. These results show that composting restructures viral communities and attenuates virus-associated ARG signals, while metagenomes and viromes provide complementary but non-interchangeable views of viral ecology and ARG risk.

## 1. Introduction

Viruses are the most abundant biological entities on Earth and can influence microbial community structure, biogeochemical cycling, biological evolution, and human health [1,2,3]. Their ecological importance has been well established in marine systems [4,5]. More recently, this recognition has extended to soils, where viruses can modulate nutrient turnover and organic matter mineralization [1,2,3]. Composting is likewise a microbially driven process of organic matter transformation, and compost products are commonly returned to soils as organic amendments [6,7]. Changes in viral communities during composting may therefore contribute to differences in the viral particles and associated genetic material, including virus-associated ARGs, introduced into soil ecosystems. Yet the succession and ecological roles of viral communities in composting systems remain insufficiently understood.

Livestock manure is an important reservoir of antibiotic residues, antibiotic resistance genes (ARGs), and potential pathogens, and composting is widely used to reduce these risks before land application [6,8,9]. Previous composting studies have mostly examined bacterial and fungal community succession alongside changes in ARGs. The thermophilic phase can attenuate ARGs in part by inactivating ARG-carrying bacterial hosts, while both temperature and thermophilic duration influence ARG reduction and possible rebound after cooling [6,10]. Shifts in ARG-carrying communities during composting may further affect the environmental persistence and dissemination of ARGs. Because viral particles can protect genetic material outside cells [11,12] and may mediate horizontal gene transfer among hosts, virus-associated ARGs are increasingly relevant to environmental ARG dissemination [13,14,15]. However, changes in viral communities and virus-associated ARGs during composting remain poorly characterized.

Environmental viral communities are commonly characterized using viromic approaches [16,17,18]. These approaches include bioinformatic identification of viral sequences from total-community metagenomes and sequencing of viral-particle-enriched fractions; here, these two data types are referred to as metagenomes and viromes, respectively. Sample processing and sequencing strategy can affect which viral populations are recovered and can therefore shape ecological interpretations of viral communities [18,19]. For example, viromes have been reported to outperform total metagenomes in characterizing viral community dynamics in agricultural soils [20]. By contrast, a recent analysis of 60 paired viromes and mixed-community metagenomes from human gut, soil, freshwater and marine environments showed that the two approaches capture different components of viral communities [19]. Viromes generally recovered higher viral species richness but lower genome abundance, whereas metagenomes retained many viral genomes missed by viromes [18,19]. Because virus-associated ARGs are usually inferred through downstream screening of metagenome or virome datasets, the approach used may also influence risk estimates. This concern is especially important in composting, a dynamic aerobic decomposition process marked by thermophilic heating, maturation and rapid physicochemical changes. These changes can drive distinct temporal changes in free viral particles, host-associated prophages, bacterial cells and extracellular ARG-bearing DNA, which are covered differently by metagenomes and viromes. The two approaches may produce different estimates of viral succession and ARG risk. Therefore, paired analysis is needed not only to catalogue viruses but also to evaluate the reliability of virus-associated ARG inference.

In this study, we used paired metagenome and virome datasets to investigate DNA viral communities and virus-associated ARGs during chicken manure composting. Here, “virus-associated ARGs” refers to putative ARG signals associated with viral sequences based on bioinformatic analysis, rather than experimentally confirmed virus-encoded ARGs. We hypothesized that composting would reshape viral community structure and reduce virus-associated ARG signals, and that the metagenome and virome approaches would recover different viral and ARG profiles by capturing partly different biological fractions. The specific objectives were to: (1) characterize changes in viral community by examining DNA viral composition and eukaryotic viral protein signals; (2) assess the environmental risk of virus-associated ARGs based on abundance, richness, and risk ranks; and (3) compare the two approaches in their recovery of viral communities and interpretation of virus-associated ARGs.

## 2. Materials and Methods

### 2.1. Compost Design and Sample Collection

The composting experiment was conducted outdoors in the summer of 2024 with three replicate windrows. Each windrow was placed under a rain shelter to prevent direct rainfall. The chicken manure (from laying hens), rice husks, and corn straw were thoroughly mixed at a ratio of 20:4:1 (*w*/*w*) and shaped into windrows. An aeration system was installed at the base of each windrow to provide periodic forced ventilation. The initial C/N ratio of the composting mixture was approximately 30, and the initial moisture content was approximately 60%. No additional water was added during composting. The composting process lasted 41 days, and the windrows were manually turned every 5 days. The core temperature of each windrow and the ambient temperature were recorded daily using temperature loggers. Compost moisture content was determined every 2 days using the oven-drying method. Sampling time points were selected based on the monitored temperature trajectory to represent the major composting stages. Raw chicken manure was collected as the initial material sample and designated as CM. Compost samples were collected on days 1 (D1), 6 (D6), 11 (D11), and 41 (D41), representing the heating, thermophilic, cooling, and mature stages, respectively. Samples from each replicate windrow were obtained by homogenizing materials collected from the upper, middle, and lower sections. The collected samples were immediately transported to the laboratory and stored at −80 °C for subsequent metagenomic DNA and viral DNA extraction.

### 2.2. Metagenomic DNA Extraction and Sequencing

Metagenomic DNA was extracted from raw chicken manure and compost samples using the ALFA Soil DNA Extraction Kit (Guangdong Magigene Biotechnology Co., Ltd., Guangzhou, China). DNA quality and concentration were assessed using a NanoDrop One spectrophotometer (Thermo Fisher Scientific, Waltham, MA, USA), a Qubit 4.0 fluorometer (Life Technologies, Carlsbad, CA, USA), and 1.5% agarose gel electrophoresis. Sequencing libraries were then constructed using the ALFA-SEQ DNA Library Prep Kit (Guangdong Magigene Biotechnology Co., Ltd., Shenzhen, China). After library quality assessment, paired-end sequencing (PE150) was performed on an Illumina NovaSeq 6000 platform (Illumina, San Diego, CA, USA), with a target sequencing depth of approximately 10 Gb per sample. 

### 2.3. Viral Particle Enrichment and Virome Sequencing

Viral particles were enriched from raw chicken manure and compost samples following previously described protocols with minor modifications [21,22]. Briefly, 10 g (dry weight) of each sample was suspended in 100 mL of 1% citrate in phosphate buffer (pH 7.0), homogenized using a high-speed tissue homogenizer for 10 s, incubated on ice for 1 min, and this procedure was repeated three times. The suspension was centrifuged at 3500× *g* for 4 min at 4 °C to remove large particles, and the supernatant was further centrifuged at 10,000× *g* for 20 min at 4 °C. The resulting supernatant was filtered through a 0.22 μm membrane to obtain viral particle suspensions, which were then concentrated using Amicon Ultra-15 centrifugal filter units (100 kDa, MilliporeSigma, Burlington, MA, USA). The concentrated suspensions were treated with DNase to remove free DNA, and viral DNA was extracted using the DNeasy PowerSoil Pro Kit (Qiagen, Hilden, Germany) according to the manufacturer’s instructions. Whole-genome amplification was then performed using the Illustra Ready-To-Go GenomiPhi V3 DNA Amplification Kit (GE Healthcare, Chicago, IL, USA).

The amplified products were quality-checked as described above. Library construction, quality assessment, and sequencing were then performed using the same procedures as those applied to the metagenomic samples. 

### 2.4. Bioinformatic Analysis

**Figure 1:**
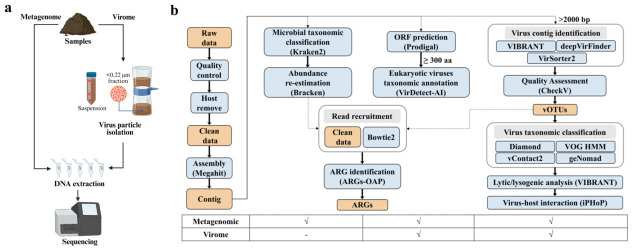
Sample processing and bioinformatics analysis pipeline for the metagenome and virome datasets. (**a**) Sample processing workflow. (**b**) Bioinformatics analysis workflow. Arrows indicate workflow progression; √ indicates steps applied to each dataset, whereas - indicates steps not applied. Abbreviations: ORF, open reading frame; ARG, antibiotic resistance gene; vOTUs, viral operational taxonomic units.

The overall bioinformatics workflow is summarized in Figure 1. Raw reads were trimmed to remove Illumina adapters and low-quality sequences using Trimmomatic v0.39 [23] with the parameters PE illuminaclip, 2:30:10 leading:15 trailing:15 slidingwindow:4:15 minlen:75. Host reads were then removed by mapping the filtered reads to the laying hen (*Gallus gallus*) reference genome (GCF_016700215.2) using BWA v0.7.17 [24] and SAMtools v1.15.1 [25]. Unmapped reads were retained as clean reads. Clean reads were assembled into contigs using MEGAHIT v1.2.9 (--k-min 21 --k-max 141 --k-step 12) [26]. For metagenomic samples, clean reads from the three biological replicates within each sampling group were pooled before assembly to improve viral contig recovery. Open reading frames were predicted using Prodigal v2.6.3 [27]. Protein sequences longer than 300 amino acids were extracted using SeqKit v2.3.1 [28] and used for eukaryotic viral protein identification with VirDetect-AI [29]. VirDetect-AI classifies input protein sequences using a deep learning framework based on convolutional and residual neural networks.

Viruses were identified from contigs longer than 2000 bp in the metagenomic and virome assemblies using a modified ViWrap pipeline v1.3.1 [30] in run_wo_read mode with the --virome option. Viral contigs were identified using VIBRANT v1.2.1 [31], VirSorter2 v2.2.3 [32], and DeepVirFinder v2020.11.21 [33], all implemented in ViWrap. The union of viral contigs predicted by the three tools was retained for downstream analyses. The identified viral contigs were clustered at 95% average nucleotide identity with at least 80% coverage using MMseqs2 v14 [34], generating viral operational taxonomic units (vOTUs). Viral genome quality and completeness were evaluated using CheckV v1.0.1 [35]. Viral sequences were clustered at the genus level using vConTACT2 v0.11.0 [36] based on gene-sharing networks with default parameters, and species-level clustering was performed using dRep v3.4.0 [37] with an ANI threshold of 95%. Viral taxonomy was assigned by integrating results from NCBI RefSeq viral protein database searches [38], VOG HMM marker searches [39], and vConTACT2 clustering. For sequences that could not be directly assigned, taxonomy was further inferred using genus-level lowest common ancestor assignment based on annotated members within the same genus cluster. Viral lifestyle (lytic or lysogenic) was predicted based on results generated by VIBRANT v1.2.1, and virus–host interactions were inferred using iPHoP v1.3.3 [40] with the default database. Finally, viral abundance was independently estimated by mapping clean reads to viral contigs using CoverM v0.6.1 [41] with the RPKM method (reads per kilobase of exon per million reads mapped).

For microbial classification of the metagenome datasets, contigs were taxonomically annotated using Kraken2 v2.17.1 [42] with Bracken v3.1 [43] against the Kraken2 standard database (k2_standard_20240112). Viral contigs identified from the metagenomic and virome datasets were mapped to their corresponding clean reads to obtain virus-associated read sets. Based on microbial taxonomic annotations, contigs classified as unclassified or viral were removed. The remaining bacterial, archaeal, and fungal contigs were mapped to clean reads to obtain microbe-associated read sets. These read sets, together with the total clean reads, were used for ARG identification using the ARGs-OAP pipeline v3.2.4 [44] with the SARG database v3.0 under default parameters. ARG abundance was normalized as PPM (parts per million), defined as the number of ARG-like reads per million mapped reads. To quantify virus-associated ARG signals, *H_total_* was defined as the total number of unique ARG-like reads detected in the clean read set, and *H_virus_* as the subset detected in the virus-associated read set. The virus-associated proportion was calculated as *H_virus_*/*H_total_*. ARG risk ranks were assigned by manually matching ARG subtype names to the published ARG risk ranking table [44,45].

All analyses were performed using default parameters unless otherwise specified. Data visualization was performed in R v4.5.0 using packages including ggplot2 v4.0.1, ComplexHeatmap v2.26.0, and circlize v0.4.17.

## 3. Results and Discussion

### 3.1. Composting Drives Temporal Succession of Viral Communities

Windrow temperature increased rapidly after composting started, reached the thermophilic phase by day 3, declined after day 8, and gradually approached ambient temperature by the end of composting (Figure S1). Moisture content decreased gradually throughout the process. Thus, the selected sampling time points represented the main composting stages: D1, D6, D11, and D41 corresponded to the heating, thermophilic, cooling, and mature stages, respectively.

A total of 32,120 putative viral contigs longer than 2000 bp were recovered from the metagenomic assemblies, yielding 27,788 vOTUs after clustering. Of these, 93.8% were predicted to be lytic. CheckV classified only small fractions of the recovered vOTUs as complete (0.1%), high-quality (0.2%), or medium-quality (0.7%), whereas 52.5% were classified as low-quality and 46.5% remained not determined. The predominance of predicted lytic viruses is consistent with previous evidence that viral lysis contributes to bacterial turnover during composting [46]. However, this proportion should be interpreted cautiously because most vOTUs were incomplete. Fragmented viral genomes may lack detectable lysogeny-associated genes, leading to underestimation of temperate viruses [47]. Taxonomic annotation was obtained for 6328 vOTUs (22.8%), although many could not be resolved across all taxonomic ranks. This low annotation rate was partly due to the inclusive viral contig identification strategy and the limited representation of environmental viral diversity in current reference databases [48,49]. Among the annotated vOTUs, *Uroviricota* was the dominant phylum (95.3%), whereas *Nucleocytoviricota* accounted for 2.78% (Table S1). *Uroviricota* mainly comprises dsDNA viruses infecting bacteria and archaea, and commonly dominates taxonomically assigned viral communities in environmental datasets [14,50,51,52]. *Nucleocytoviricota* includes large and giant dsDNA viruses infecting diverse eukaryotic hosts and is increasingly recognized as an underexplored component of both aquatic and terrestrial viral communities [53,54,55]. Its detection here suggests that this viral group may have underrecognized ecological roles in organic waste treatment systems.

**Figure 2:**
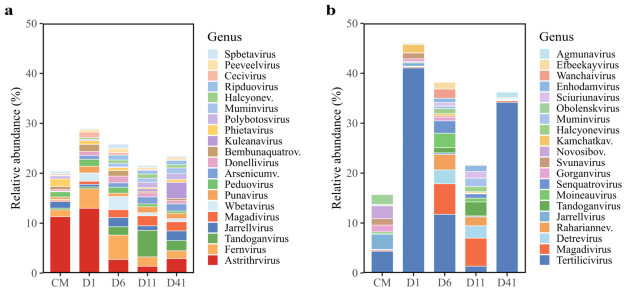
Relative abundance of the top 20 viral genera during chicken manure composting. (**a**) Viral genera recovered from the metagenome dataset. (**b**) Viral genera recovered from the virome dataset. CM represents raw chicken manure, while D1, D6, D11, and D41 represent samples collected on days 1, 6, 11, and 41 of composting, respectively.

The viral community recovered from the metagenome dataset showed clear temporal succession during composting (Figure 2a), with 620 genera identified. Several genera that were relatively abundant in raw chicken manure, including *Astrithrvirus*, *Phietavirus*, and *Peduovirus*, generally declined over time. This trend was particularly evident for *Astrithrvirus*, whose relative abundance decreased from 11.31% at CM to 1.37% at D11. The decline of these initially abundant genera indicates that the changing conditions during composting progressively filtered the manure-associated viral community [56]. In contrast, several genera showed stage-dependent enrichment. *Fernvirus* was enriched during the early and thermophilic stages, *Tandoganvirus* increased and reached its highest proportion at the cooling stage (5.30%), and *Kuleanavirus* was enriched at the end of composting. These contrasting patterns indicate that composting selectively restructured the viral community rather than causing a uniform decline in viral abundance. This restructuring may have resulted from the combined effects of thermal stress, host turnover, and reduced survival of manure-associated viral particles [46,56].

The virome dataset yielded 31,507 putative viral contigs longer than 2000 bp, which were clustered into 26,271 vOTUs. Predicted lytic vOTUs dominated the virome dataset (96.0%), consistent with the metagenome dataset. Although the virome recovered fewer vOTUs, it yielded more taxonomically assigned vOTUs (9943; 37.8%) and a higher proportion of relatively complete genomes (2.5% vs. 1.0%). The higher annotation rate and genome completeness indicate improved recovery of particle-associated viral genomes. This pattern is consistent with a recent cross-environment comparison showing that viromes generally recover more complete viral genomes than paired metagenomes, although the magnitude of this difference varies among environments [19]. However, this advantage does not imply a more comprehensive representation of the compost viral community, because virome preparation can selectively affect the recovery of different viral groups [57]. Among the annotated vOTUs, *Uroviricota* was dominant (90.7%), followed by *Hofneiviricota* (6.6%). Within *Hofneiviricota*, *Inoviridae* was the most abundant family (Table S2). Members of this family are filamentous viruses with circular positive-sense ssDNA genomes [58,59]. Their high abundance in the virome may partly reflect amplification bias, as whole-genome amplification preferentially amplifies circular ssDNA templates [57,60,61,62].

The virome also exhibited clear temporal succession during composting, but its genus-level composition was more strongly dominated by a few taxa than that of the metagenome dataset (Figure 2b). After excluding vOTUs without genus-level assignments, a total of 675 genera were identified. Several genera that were relatively abundant in raw chicken manure, including *Jarrellvirus*, *Novosibovirus*, and *Obolenskvirus*, decreased as composting progressed. Other genera displayed stage-specific enrichment. *Tertilicivirus* dominated most sampling points, reaching particularly high relative abundances at D1 (41.08%) and D41 (34.19%). Because *Tertilicivirus* belongs to *Inoviridae*, its apparent dominance may partly reflect preferential amplification of circular ssDNA genomes during whole-genome amplification [60]. Comparison of the overall genus-level profiles showed that the dominant genera differed substantially between the two datasets. Only a few temporal patterns were shared, including increases in *Magadivirus* and *Tandoganvirus*. These contrasting profiles likely reflect both the partly different biological fractions captured by the two approaches and their distinct analytical workflows. Metagenomes recover viral DNA from the total community, whereas viromes target a particle-enriched fraction and are additionally influenced by the enrichment and amplification procedures. Similar method-dependent differences have been reported in paired comparisons across environments [19], supporting the view that the two datasets provide complementary rather than interchangeable representations of compost viral community succession.

### 3.2. Composting Reduces Eukaryotic Viral Protein Signals

Eukaryotic viruses are less frequently examined than prokaryotic viruses in environmental studies based on DNA sequencing. In manure composting systems, viral signals associated with animal or human hosts may provide indirect information on potential biological risks and their reduction during treatment. VirDetect-AI was used to screen predicted protein sequences from both the metagenome and virome datasets, and eukaryotic viral signals were evaluated at the protein level.

**Figure 3:**
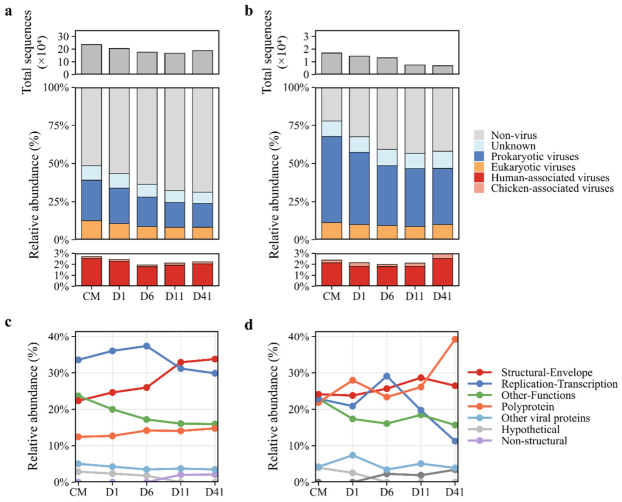
Viral protein signals and predicted human-host-associated functions during chicken manure composting. (**a**) Total protein sequences and VirDetect-AI classification proportions in the metagenome dataset. (**b**) Total protein sequences and VirDetect-AI classification proportions in the virome dataset. (**c**) Functional composition of predicted viral protein sequences associated with human and chicken hosts in the metagenome dataset. (**d**) Functional composition of predicted viral protein sequences associated with human and chicken hosts in the virome dataset.

In the metagenome dataset, 1.67–2.36 × 10^5^ predicted proteins per sample were screened (Figure 3a). More than 60% were classified as non-viral or unknown, while the proportion classified as viral decreased during composting. Eukaryotic viral proteins followed the same trend and accounted for 8.15–12.45% of all sequences. This reduction may partly reflect loss of viral particle integrity and viral genome degradation under the thermal and biologically active conditions [46]. Sequences assigned to viruses associated with *Homo sapiens* and *Gallus gallus* represented 2.14% and 0.17% of all classified protein sequences, respectively. These viral protein sequences decreased in number and shifted in functional composition during composting (Figure 3c). Replication-transcription proteins dominated the early stages (D1 and D6), whereas structural-envelope proteins were most represented at later stages (D11 and D41). These categories are mainly associated with viral genome replication and expression, and with virion structure and host interaction, respectively [63,64]. Although these results do not directly measure viral activity or infectivity, the decline in human-associated eukaryotic viral protein signals suggests that composting may reduce the potential risk associated with human viruses. The decrease in signals related to replication also supports this interpretation. Previous studies have also provided more direct evidence that manure composting can reduce the genome abundance and infectivity of human enteric viruses, although the extent of reduction varies among virus types [65].

The virome dataset contained fewer than one-tenth as many input protein sequences as in the metagenome dataset. However, viral proteins accounted for a much larger proportion of the classified sequences, reaching 67.7% in individual samples and approximately twice the proportion observed in the corresponding metagenome (Figure 3b). This higher proportion was driven primarily by prokaryotic viral proteins. This difference reflects the distinct sequence backgrounds captured by the two approaches. The metagenome retained a broader non-viral sequence background [20], whereas the virome preferentially recovered viral genomes. Despite this difference, the proportions of eukaryotic viral proteins, including those associated with *Homo sapiens* or *Gallus gallus*, differed only slightly between the two datasets. This similarity may reflect the contribution of intracellular, integrated, or nonencapsidated eukaryotic viral DNA to the metagenome, or the reduced recovery of large eukaryotic viral particles from the virome during 0.22 μm filtration [66,67]. At the functional level, however, the two datasets differed, polyproteins became the dominant functional category in the corresponding virome subset (Figure 3d). These proteins are initially translated as a single precursor polypeptide and subsequently cleaved into multiple mature viral proteins [68,69]. Their predominance may reflect differences in the viral taxa represented by the two datasets and the greater recovery of relatively complete viral coding regions in the virome.

### 3.3. Composting Reduces Virus-Associated ARG Signals and Their Risk Ranks

**Figure 4:**
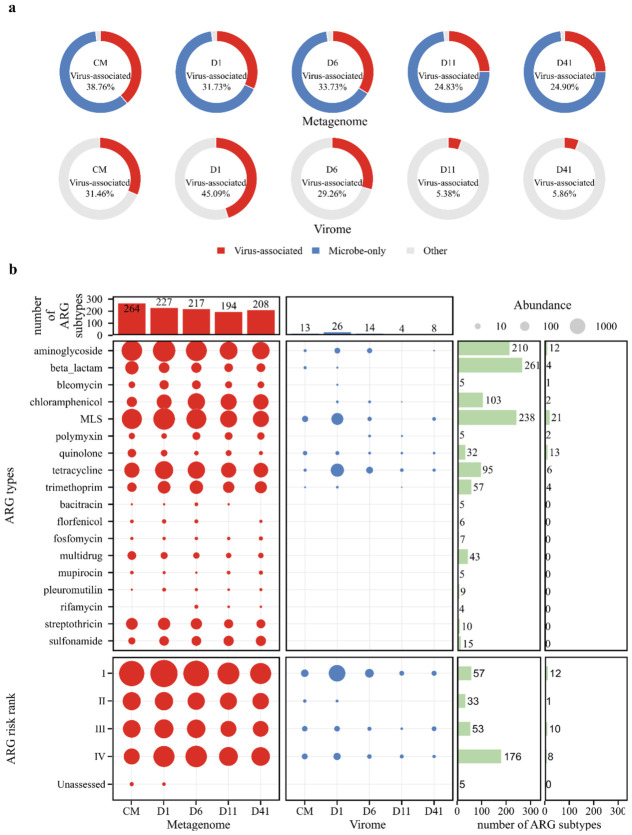
Composition, abundance, and risk rank distribution of virus-associated ARGs during chicken manure composting. (**a**) Proportions of virus-associated ARGs in total ARGs from the metagenome and virome datasets. (**b**) Virus-associated ARG abundance and subtype richness in the metagenome and virome datasets, grouped by ARG type and risk rank. Upper bar plots show the number of ARG subtypes in each sample, bubble plots show ARG abundance expressed as PPM, and right bar plots show the number of ARG subtypes assigned to each ARG type or risk rank.

In the metagenome dataset, 24 ARG types and 883 subtypes were detected, including 18 types and 324 subtypes in the virus-associated fraction. Across samples, virus-associated ARGs accounted for 24.90–38.76% of total ARG abundance, while most ARG abundance was assigned to the microbial fraction (Figure 4a). This pattern is consistent with previous studies showing that bacteria are the primary ARG carriers in manure and compost metagenomes [70,71]. Aminoglycoside, MLS, tetracycline, and chloramphenicol resistance genes were the dominant ARG types in the virus-associated fraction (Figure 4b). These ARG types are also commonly found at high abundance in chicken manure and other livestock wastes, reflecting the manure resistome shaped by antibiotic exposure and persistent bacterial hosts [72,73]. Their predominance in the virus-associated fraction may therefore partly reflect the overlap in dominant ARG types between the virus-associated and microbial fractions. During composting, the proportion of virus-associated ARG signals decreased by 13.86 percentage points (Figure 4a), indicating that composting attenuated the viral-sequence-associated resistome. The abundance of most ARG types increased transiently during the initial stage and then declined toward the end of composting (Figure 4b), whereas beta-lactam and multidrug ARGs declined continuously throughout the process. Virus-associated ARG richness also decreased by 21.21%, mainly due to the loss of beta-lactam resistance subtypes. The early increases may reflect rapid restructuring of ARG-carrying host and viral communities during initial heating, potentially accompanied by a temporary increase in detectable ARG-containing DNA following host lysis or prophage induction [46,74]. The subsequent decline may be partly associated with the loss of ARG-carrying hosts during thermophilic treatment, because both temperature and the duration of thermophilic exposure are important factors controlling ARG attenuation and transfer potential [6,10,75]. In contrast, the abundance of polymyxin resistance genes increased by the end of composting, possibly reflecting the persistence or enrichment of host taxa carrying these genes [75].

In the virome dataset, 20 ARG types and 330 subtypes were detected, including 9 types and 31 subtypes in the virus-associated fraction. Compared with the metagenome dataset, the virome dataset showed substantially lower virus-associated ARG abundance and richness (Figure 4a,b). This difference may be related to the low nucleic acid yield during virome preparation and to the fact that ARGs are rarely encoded in phage genomes [76,77]. Tetracycline and MLS ARGs dominated the virus-associated ARG profile in the virome dataset, consistent with the metagenomic results. As observed in the metagenome dataset, virus-associated ARG abundance and richness declined overall in the virome, whereas their proportion within the total ARG signal varied more markedly across composting stages (Figure 4b). Notably, the abundance of *tet(L)*, *cfr(A)*, and *erm(T)* increased sharply at D1, reaching up to 1260-fold the levels detected in CM, and then declined rapidly at later stages. These short-lived increases in the particle-enriched viral fraction may have been associated with rapid turnover of ARG-carrying hosts and their associated viral populations [46,74]. The large fold changes may also be partly attributable to the very low abundances in CM and bias introduced during whole-genome amplification [57,60]. At the same time, these genes have frequently been reported in livestock manure and manure-derived organic fertilizers, where their high abundances are commonly associated with persistent bacterial carriers, particularly tolerant *Bacillota* taxa such as *Bacillales* [78,79,80]. Their detection within the virus-associated ARG fraction suggests that viruses may also contribute to their persistence. By contrast, some ARG categories, particularly aminoglycoside resistance genes, were less prominent in the virome dataset than in the metagenome dataset. This discrepancy may reflect ARG signals associated with prophages, free phage DNA fragments, or intracellular replication intermediates [81,82] that are captured by metagenomes but are not present in viral-particle-enriched virome [83]. The broader DNA background and imperfect bioinformatic separation of viral and cellular sequences may also contribute to overestimation of virus-associated ARG signals in the metagenome dataset [18,76]. Thus, the two datasets should be regarded as capturing overlapping but differently weighted pools of virus-associated ARG signals, rather than as equivalent measurements of the same viral ARG fraction.

Virus-associated ARG subtypes were further assigned to risk ranks based on mobility, human association, and occurrence in potential pathogenic hosts [45]. In the metagenome dataset, Rank I ARGs dominated the abundance profile, whereas Rank IV ARGs contained the largest number of subtypes (Figure 4b). This contrast indicates that a relatively small number of high-risk ARG subtypes accounted for a large proportion of the virus-associated ARG signal. Rank I ARGs also dominated the virome dataset, although the overall risk-ranked ARG signal was weaker. Despite differences between the two datasets, both showed a clear reduction in virus-associated ARG risk signals during composting.

### 3.4. Predicted Virus–Host Associations Suggest Possible Phage-Mediated Gene Exchange

**Figure 5:**
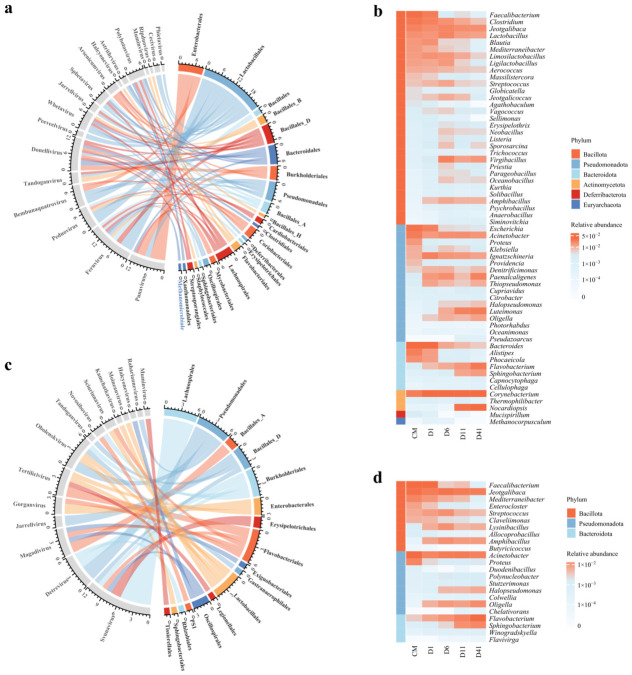
Predicted virus–host linkages and host abundance patterns during chicken manure composting. (**a**) Predicted linkages between the top 20 viral genera and bacterial host genera in the metagenome dataset. (**b**) Abundance patterns of bacterial hosts linked to metagenome-derived viral genera. (**c**) Predicted linkages between the top 20 viral genera and bacterial host genera in the virome dataset. (**d**) Abundance patterns of bacterial hosts linked to virome-derived viral genera. In panels (**a**) and (**c**), gray labels on the left indicate viral genera, while black and blue labels on the right indicate bacterial and archaeal host genera, respectively. In panels (**b**) and (**d**), the colored bars beside the heatmaps indicate bacterial phyla, whereas the heatmap color scale represents Bracken-estimated relative abundance on a log10 scale.

Phage-mediated transduction can facilitate horizontal transfer of antibiotic resistance genes among hosts [84,85]. To characterize virus–host associations relevant to possible gene exchange, putative hosts of vOTUs were predicted using iPHoP v1.3.3. In the metagenome dataset, the 20 most abundant viral genera were linked to 114 host genera, of which 60 (52.6%) were also detected in the metagenome-derived microbial dataset (Figure 5a). Among these 60 genera, one belonged to Archaea and the remaining 59 belonged to Bacteria, mainly *Bacillota*, *Pseudomonadota*, and *Bacteroidota* (Figure 5b). The predominance of these phyla among the predicted hosts was consistent with the bacterial composition of the compost [86,87,88]. This further suggests that changes in host availability and community composition may have contributed to the temporal succession of viral communities during composting [46,56]. Of the 59 bacterial host genera, 22 were flagged as potentially pathogenic based on strain-level records in BacDive (Table S3), including *Acinetobacter*, *Escherichia*, *Klebsiella*, and *Listeria*. *Bacteroides*, *Phocaeicola*, and *Capnocytophaga* also merit attention because some members are commensals in their usual niches but can act as opportunistic pathogens under favorable conditions [89,90,91]. Among these potentially pathogenic genera, *Bacteroides* and *Phocaeicola* were each linked to multiple viral genera. Conversely, *Peduovirus*, *Fernvirus*, and *Donellivirus* showed relatively broad predicted host ranges. Although these predicted links do not confirm active infection or transduction, they indicate potential opportunities for virus-mediated gene exchange [82,92].

In the virome dataset, 15 of the 20 most abundant viral genera were linked to 46 host genera, of which 24 (52.2%) were detected in the microbial dataset (Figure 5c,d). All matched hosts belonged to Bacteria, and eight were flagged as potentially pathogenic (Table S4), including *Acinetobacter*, *Streptococcus*, and *Proteus*. *Svunavirus* and *Detrevirus* showed the broadest predicted host ranges. Although fewer virus–host linkages were predicted from the virome, a similar proportion of predicted hosts was detected in the microbial dataset (52.2% vs. 52.6%). Among these matched bacterial hosts, the proportion of genera containing potentially pathogenic strains was also comparable between the virome and metagenome datasets (33.3% vs. 37.3%). The nearly identical host-matching rates provide some indirect support for the reliability of the predictions [40]. The remaining mismatches may reflect hosts below the detection limit of microbial profiling, incomplete reference genome coverage, or uncertainty in computational host prediction [40,93]. Thus, the smaller virome-derived host network likely reflects differences in the viral sequences recovered for host prediction rather than a genuinely narrower range of hosts.

Across the two datasets, predicted links between abundant viral genera and bacterial hosts such as *Bacteroides*, *Phocaeicola*, *Corynebacterium*, *Listeria* and *Acinetobacter* identify plausible ecological interfaces where viral turnover could intersect with clinically relevant bacteria. However, because host predictions do not establish infection or transduction, these links should be presented as hypotheses for future validation rather than direct evidence of ARG transfer. More broadly, the ecological significance of the observed viral dynamics and virus-associated ARG signals ultimately depends on their links to host physiology, fitness, and ecological performance [94]. Further studies linking viral genetic composition with host phenotypic responses would help clarify the consequences of virus-associated genes for microbial interactions and ecological processes during composting.

## 4. Conclusions

Chicken manure composting acted as a strong ecological filter on the manure viruses, replacing parts of the initial manure-associated viral assemblage with stage-specific viral populations and reducing virus-associated ARG abundance, richness, and risk signals by maturity. Importantly, paired metagenome and virome analyses revealed broadly consistent trends (viral community succession and ARG reduction) but dataset-specific viral signatures (e.g., dominant viral taxa and the diversity and abundance of virus-associated ARGs) during composting. These findings also show that estimates of virus-associated ARGs require cautious interpretation because metagenomes and viromes capture different viral fractions and are affected differently by contamination, enrichment, and amplification. Future work combining long-read viral genomics, infectivity assays, prophage induction measurements, and experimentally validated host linkages will be necessary to determine whether the detected ARGs are truly packaged, transferable, and biologically active.

## Data Availability

The raw sequencing data generated in this study are publicly available in the ScienceDB (https://doi.org/10.57760/sciencedb.41127; accessed on 15 July 2026).

## Supplementary Materials

The following supporting information can be downloaded at: https://www.mdpi.com/article/10.3390/v18070789/s1, Figure S1: The temperature and moisture of composting in the full-scale experiment; Table S1: Taxonomic assignments and RPKM-normalized abundance of metagenome-derived vOTUs across composting stages; Table S2: Taxonomic assignments and RPKM-normalized abundance of virome-derived vOTUs across composting stages; Table S3: BacDive-based potential pathogenicity categories of predicted hosts for metagenome-derived viral genera; Table S4: BacDive-based potential pathogenicity categories of predicted hosts for virome-derived viral genera.
